# Cardiolipin’s multifaceted role in immune response: a focus on interacting proteins

**DOI:** 10.3389/fimmu.2025.1680326

**Published:** 2025-12-08

**Authors:** Zhuodong Chai, Yuqi Zhou, Sukria Hossain, Khanh Huynh, Sahelosadat Hajimirzaei, Yan Zhang, Jiaqian Qi, Guoying Zhang, Zhenyu Li, Yinan Wei

**Affiliations:** Department of Pharmaceutical Sciences, Irma Lerma Rangel School of Pharmacy, Texas A&M University, College Station, TX, United States

**Keywords:** inflammasome, cardiolipin, protein-lipid interaction, innate immunity, pyroptosis

## Abstract

Cardiolipin is a unique and essential phospholipid that plays a pivotal role in cellular function. In eukaryotic cells, it is predominantly localized within the mitochondrial membranes, with the highest concentration in the inner mitochondrial membrane (IMM). Recent studies have highlighted the multifaceted role of cardiolipin in immune regulation. This review aims to provide a comprehensive overview of specific proteins that directly interact with cardiolipin and to elucidate how these interactions underlie its diverse and critical functions in innate immunity. In addition, we discuss the involvement of cardiolipin in various pathological conditions in which its protein interactions contribute to immune dysregulation.

## 1 Cardiolipin as a central player in immunometabolism

### 1.1 Structure, localization, and fundamental mitochondrial functions

**Figure 1:**
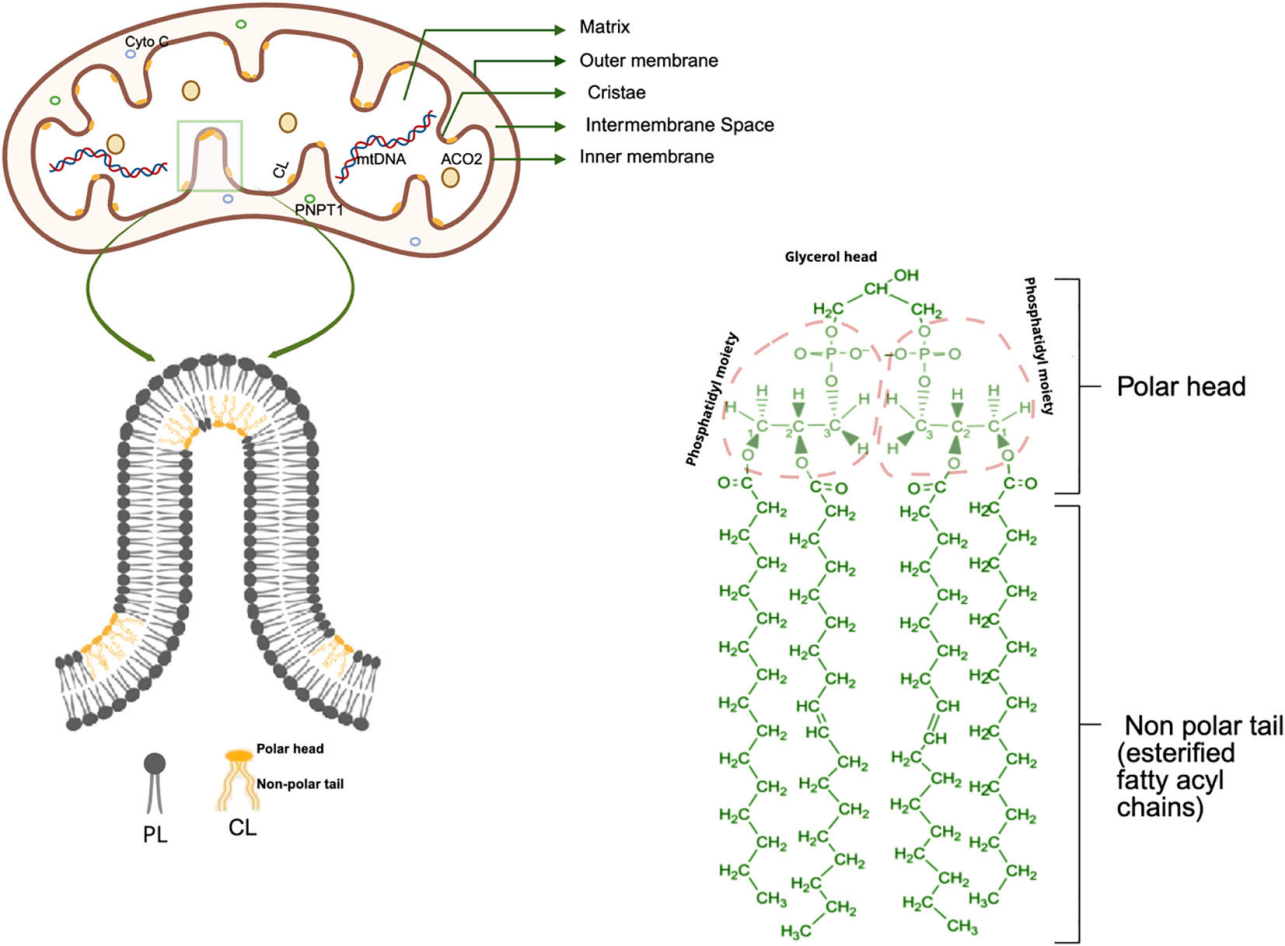
Structural features of cardiolipin. cardiolipin (CL) possesses a central glycerol backbone that is linked to two phosphatidyl residues, forming an anionic polar head, giving cardiolipin a conical geometry. Such a structure enables cardiolipin to generate lateral pressure within the lipid bilayer, thereby facilitating the induction of negative curvature and contributing to the formation of highly curved domains within the IMM (6, 9).

Cardiolipin accounts for approximately 15–20% of the total mitochondrial phospholipid content (1). Its distinctive dimeric structure, characterized by four acyl chains and two phosphatidyl moieties linked to a central glycerol backbone, confers a conical shape that is critical for its ability to induce membrane curvature (**Figure 1**) (1, 2). Cardiolipin contributes to the formation of mitochondrial cristae, as the deficiency of cardiolipin leads to disintegration of mitochondrial cristae and disruption of membrane structures (3). This also explains why cardiolipin mainly accumulates in the IMM, with over half of it residing in the inner leaflet (4, 5). The composition of the acyl chains in cardiolipin is tissue-specific. For example, in the brain, cardiolipin usually contains acyl chains derived from arachidonic acid (AA, 22:4) and docosahexaenoic acid (DHA, 22:6), while in the heart and liver, linoleoyl chains derived from linoleic acid (LA, 18:2) are more common (6, 7). The structural and positional asymmetry of cardiolipin renders it a key regulatory molecule in the process of cell death. When mitochondrial function is impaired, cardiolipin translocates to the outer membrane or gets released into the surrounding environment, promoting inflammatory responses and/or other cellular pathways (8).

The biosynthesis, remodeling, assembly, oxidation, and degradation of cardiolipin has been thoroughly reviewed (10). Briefly, immature cardiolipin is synthesized in the IMM from phosphatidylglycerol (PG) and cytidine diphosphate-diacylglycerol (CDP-DAG), catalyzed by cardiolipin synthase (CRLS). It is then remodeled into mature cardiolipin containing unsaturated fatty acid side chains by phospholipase A2 (PLA2) and Tafazzin (TAZ) (11).

The presence of cardiolipin is fundamental for various vital mitochondrial functions (1, 12). It is indispensable for cellular respiration, efficient energy conversion, and the intricate process of oxidative phosphorylation (OXPHOS) (13). Cardiolipin plays a critical role in stabilizing and organizing the enzyme complexes of the electron transport chain (ETC) into higher-order assemblies, or supercomplexes, that are essential for efficient electron transport and ATP synthesis. Beyond its bioenergetic functions, cardiolipin is indispensable for maintaining mitochondrial membrane morphology and stability, as well as for regulating dynamic processes such as fusion and fission. It also contributes to mitochondrial biogenesis and facilitates protein import.

### 1.2 The emergence of cardiolipin as a danger-associated molecular pattern

The specific acyl chain composition and redox state of cardiolipin are critical for mitochondrial health (12). Pathological deviations, such as a decrease in cardiolipin levels, alterations in its fatty acyl chain composition, or excessive oxidation, can severely compromise mitochondrial function. Such derangements are associated with increased production of reactive oxygen species (ROS) and initiate broader cellular stress responses, signaling a departure from cellular homeostasis. Unsaturated fatty acid side chains in cardiolipin are prone to oxidation (forming oxidized cardiolipin), which has been associated with cardiolipin translocation to the outer membrane upon ROS stimulation. The synthetic peptide SS-31 (also known as Elamipretide) associates with cardiolipin, mitigating its pathological effects through ROS scavenging and mitochondrial membrane stabilization (14, 15), which subsequently attenuate the downstream activation of the NLRP3 inflammasome (16). In addition, Annexin A5, which binds to oxidized cardiolipin, has been shown to reduce inflammatory responses (17). On the other hand, during apoptosis, cardiolipin is oxidized by cytochrome c (Cyt c) and serves as a signal of cell damage (18).

Under physiological conditions, cardiolipin is sequestered within the IMM of healthy eukaryotic cells, effectively shielding it from the immune system, with only a small fraction presenting in the outer mitochondrial membrane (OMM) under basal conditions (6, 19, 20). In response to various forms of mitochondrial stress, damage, or cellular injury, cardiolipin’s localization can be altered. It may become exposed on OMM (externalized cardiolipin) or released into the cytosol or extracellular milieu (released cardiolipin) (11). Externalized cardiolipin could act as a potent DAMP for the immune system (20). Similarly, studies suggest that oxidized or saturated cardiolipin, which may accumulate in Barth Syndrome (21–24), could act as DAMPs. However, native unsaturated cardiolipin is not pro-inflammatory and does not induce cell death (25). This recognition is considered critical, as it enables the host immune system to detect and respond to endogenous signals of cellular distress in a manner analogous to its recognition of pathogen-associated molecular patterns (PAMPs) from invading microbes. The signaling functions of distinct cardiolipin species and their oxidation products are increasingly appreciated as potential intracellular and extracellular mediators that modulate both innate and adaptive immune responses.

The transition of cardiolipin from a structural lipid confined within the IMM to an active immune signaling molecule upon mitochondrial perturbation represents a fundamental mechanism by which cellular stress is communicated to the immune system. This phenomenon highlights cardiolipin’s role as an endogenous “alarm signal” that prompts an immune response to either restore cellular homeostasis or facilitate the elimination of compromised cells. This interplay between mitochondrial integrity and immune activation underscores the profound integration of cellular metabolism with immune surveillance.

Cardiolipin has a high affinity for various proteins, including both mitochondrial and non-mitochondrial ones, as well as large protein complexes (6, 26). Its phosphate head group, which carries two negative charges, renders it prone to nonspecific interactions through electrostatic interaction. As a result, cardiolipin mostly exists in association with a variety of proteins and multiprotein complexes (27). In mitochondria, cardiolipin induces changes in the tertiary structure and catalytic activity of proteins, promotes protein–protein interactions, assists in the assembly of large protein complexes, and participates in protein transport and signal transduction, playing a critical role in maintaining normal mitochondrial morphology and physiological functions (1, 12, 28, 29).

Dysregulation of cardiolipin metabolism and inflammasome signaling has been linked to diverse diseases such as neurodegenerative disorders, diabetic kidney disease (DKD), atherosclerosis, and cancer (6, 30–33). The altered cardiolipin remodeling and oxidation have been associated with mitochondrial injury and podocyte dysfunction in DKD (30). In neurodegenerative disorders such as Alzheimer’s and Parkinson’s diseases, cardiolipin oxidation has been shown to contribute to triggering neuronal cell death and inflammation (6). In atherosclerosis, oxidized cardiolipin may exacerbate inflammation and plaque instability (32).

## 2 Protein partners that mediating cardiolipin’s role in innate immunity

The innate immune system serves as the body’s first line of defense, rapidly recognizing and responding to conserved molecular patterns indicative of danger, including PAMPs, DAMPs, and homeostasis-altering molecular processes (HAMPs), using pattern recognition receptors (PRRs) such as the TLR receptors. One key downstream response is the formation of inflammasomes, which consist of sensors, adaptors, and effectors (34, 35). Beyond this well-established role, evidence suggests that cardiolipin, particularly when its typical mitochondrial localization is disrupted, acts as a central signaling hub by directly interacting with diverse immune-related proteins (**Table 1**). These include not only components of inflammasomes (e.g., NLRP3, caspase-1), but also mediators of apoptosis (e.g., Cyt c), autophagy and mitophagy (e.g., Light Chain 3, Beclin 1), and pattern recognition receptors (e.g., TLR2, TLR4). These interactions position cardiolipin as a critical regulator at the interface of immune sensing, mitochondrial stress, and programed cell death.

**Table 1:** Summary of key cardiolipin–protein interactions.

Interacting protein	Suggested functional role	Evidence for interaction with cardiolipin	Immunological/Cellular outcome	Key references
Cytochrome c	Anchored by cardiolipin; oxidized cardiolipin converts cytochrome c into peroxidase	Binding with cardiolipin in liposome	Initiates apoptosis via cytochrome c release and caspase activation	(36–38)
Mitophagy mediators (LC3, Beclin 1)	Recognizes cardiolipin externalized on the outer mitochondrial membrane as an “eat-me” signal	Vesicle flotation assay using cardiolipin containing liposomes	Promotes mitophagy to remove damaged mitochondria	(39, 40)
NLRP3 Inflammasome (NLRP3 + caspase-1)	Cardiolipin externalization recruits and activates NLRP3	Lipid strip binding assay	Activates caspase-1and caspase-8, leading to pyroptosis	(41, 42)
TLR families (TLR4, TLR2)	Recognizes cardiolipin as an agonist or antagonist	Competitive inhibition by cardiolipin; gene deficient cell model	Activates NF-κB signaling and production of pro-inflammatory cytokines (TNF-α, IL-6)	(43–46)
Gasdermin families	Forms pores in plasma and mitochondrial membranes upon activation	Lipid strip binding assay; gene deficient cell model	Triggers pyroptosis and mitochondrial permeabilization	(31, 47)
Pyroptosis caspases (Caspase-1/4/11)	Modulate activities of caspase-1/4/11	Competitive inhibition of Caspase-4/11 by cardiolipin; binding with cardiolipin in liposome; lipid strip binding assay	Modulates inflammatory signaling and cell death via exposed CL: potentiates caspasp-1 signal and inhibits caspase-4/11 signal	(25, 42)
Apoptosis caspases (Caspase-8)	Activates Caspase-8	Binding with cardiolipin in liposome; gene-deficient cell models	Triggers mitochondrial permeabilization, cytochrome c release, apoptosome formation, intrinsic and extrinsic apoptosis	(48–50)
Pro-IL-1α	Competes with LC3 for cardiolipin binding	Lipid strip binding assay	Impaired mitochondria promotes the activation of the NLRP3 inflammasome	(51)

### 2.1 Cyt c: from electron shuttle to apoptotic peroxidase

Cyt c is a central protein within mitochondria, performing a dual role vital for cell survival and death. In healthy cells, it functions as an electron shuttle between respiratory complexes III and IV, essential for energy production. However, under conditions of cellular stress, Cyt c can transition into a pro-apoptotic role, contributing to the initiation of programmed cell death (5).

#### 2.1.1 Molecular interaction with cardiolipin and peroxidase activity

In its native state, cytochrome c (Cyt c) contains a hexa-coordinated heme iron, which largely restricts its peroxidase activity (52). Externalization of cardiolipin to the outer leaflet of the IMM enhances its interaction with Cyt c, altering heme coordination and inducing structural destabilization and partial unfolding of the protein. This conformational change converts Cyt c from an electron carrier into a peroxidase with high activity toward cardiolipin (12, 53–55). The newly acquired peroxidase activity exhibits remarkable specificity toward cardiolipin, catalyzing the oxygen-dependent peroxidation and subsequent hydrolysis of its unsaturated acyl chains to generate oxidized cardiolipin (cardiolipin-OOH) (12). The Cyt c–cardiolipin interaction involves two distinct binding modes: one mediated by electrostatic attraction between the phosphate head group of cardiolipin and lysine residues on Cyt c, and another driven by hydrophobic interactions, wherein one or more fatty acyl chains of cardiolipin insert into a hydrophobic channel within the Cyt c molecule (36–38).

#### 2.1.2 Role in mitochondrial outer membrane permeabilization and apoptotic cell clearance

The release of cytochrome c (Cyt c) into the cytosol is a pivotal event that triggers the caspase cascade, leading to apoptosome formation and execution of programmed cell death. The accumulation of oxidized cardiolipin on the OMM serves as a critical signaling platform during apoptosis. The dynamic interaction between cardiolipin and Cyt c functions as a “redox switch” that determines cell fate. Upon binding to cardiolipin, Cyt c acquires peroxidase activity and catalyzes cardiolipin oxidation. The resulting oxidized cardiolipin exhibits reduced affinity for Cyt c, facilitating its dissociation from the IMM and subsequent release into the cytosol (1). This release represents a key step in the apoptosis process. Consequently, this feedback loop, in which cardiolipin activates Cyt c to oxidize cardiolipin, contributes to the generation and modulation of immunogenic signals, including oxidized cardiolipin. These signals can promote secondary necrosis and activate innate immune responses, particularly during the clearance of apoptotic cells (56–58). This illustrates how intracellular biochemical events can give rise to immune signaling.

### 2.2 Light chain 3 and Beclin 1: driving mitophagy for immune homeostasis

Mitophagy, a specialized form of autophagy, refers to the selective degradation and removal of damaged or superfluous mitochondria. This process is essential for maintaining mitochondrial quality and network integrity, thereby supporting cellular survival and homeostasis, particularly in response to stress, injury, or infection (39). In response to cellular stress, cardiolipin translocates from the inner to the outer mitochondrial membrane, where it functions as a potent “eat-me” signal. By serving as a binding site for components of the autophagic machinery, externalized cardiolipin initiates the process of mitophagy (12). This translocation is regarded as a key trigger for the selective clearance of damaged mitochondria. On the outer mitochondrial membrane, cardiolipin directly interacts with LC3, a core autophagy protein essential for autophagosome formation and cargo recruitment (39, 59, 60).

Studies indicate that LC3A and LC3B interact with cardiolipin-containing membranes, with LC3A demonstrating a stronger binding affinity compared to LC3B (39). These interactions display high specificity for cardiolipin compared with other negatively charged phospholipids. Upon cardiolipin externalization, both LC3A and LC3B colocalize with mitochondria, and simultaneous silencing of these proteins markedly reduces mitophagy, underscoring their important role in cardiolipin-mediated mitochondrial clearance. Interestingly, LC3A may also recognize oxidized cardiolipin, which could help prevent its recognition by pro-apoptotic factors and thereby mitigating excessive apoptosis. In contrast, LC3C, although exhibiting stronger cardiolipin binding *in vitro*, shows lower specificity, and its mitochondrial colocalization is not stress-dependent—suggesting a distinct role in maintaining mitochondrial homeostasis rather than directly initiating mitophagy.

Beclin 1 is a pivotal protein within the autophagic core machinery (61). A conserved domain of Beclin 1 has been shown to directly bind to cardiolipin on the OMM (40). This interaction can either promote or inhibit mitophagy, indicating a complex regulatory role for Beclin 1 in fine-tuning the autophagic response to mitochondrial signals (40).

Mitophagy is not merely a cellular housekeeping mechanism; it is critically important for maintaining homeostasis within the innate immune system (62). The interaction of externalized cardiolipin with LC3 and Beclin 1 to initiate mitophagy represents an essential cellular quality control mechanism that directly impacts immune regulation. By clearing damaged mitochondria, this process prevents the chronic release of pro-inflammatory DAMPs and the subsequent aberrant immune activation, thereby contributing to the maintenance of immune homeostasis. This highlights mitophagy as an important anti-inflammatory quality control mechanism (63).

### 2.3 Toll-like receptors, sensors for innate immunity

Toll-like receptors (TLRs) are an important class of pattern recognition receptors in the innate immune system, capable of recognizing PAMPs and activating immune responses. TLR4 is a key pattern recognition receptor for detecting lipopolysaccharide (LPS) from Gram-negative bacteria. TLR4, together with co-receptor MD-2, forms a recognition complex that mediates downstream signal transduction, while CD14 helps to transfer LPS to the complex. TLR2 recognizes microbial membrane components such as lipoteichoic acid and other lipoproteins from bacteria (64, 65). Both can activate the transcription factor NF-κB, and provide the “first signal” (priming) for NLRP3 inflammasome activation (66).

#### 2.3.1 TLR4

The lipid A moiety determines the immunostimulatory activity of LPS (67). The phosphate groups of lipid A are important for the agonistic activity, as deletion of either phosphate group reduces the endotoxic activity (68). The number and length of acyl chains are among the major determinants influencing the agonistic activity of lipid A (69–71). For example, the hexa-acylated lipid A of *Escherichia coli* functions as a potent TLR4 agonist across mammalian species. In contrast, its tetra-acylated precursor, lipid IVa, exhibits species-specific activity, acting as a weak agonist in mice but as an antagonist in humans (72). TLR4 requires the coreceptor MD-2 to bind with LPS (73), while MD-2 can directly bind to LPS in its hydrophobic cavity with high affinity (74). Binding of LPS to the MD-2/TLR4 complex leads to dimerization and activation of the TLR4 signaling pathway (24, 45).

**Figure 2:**
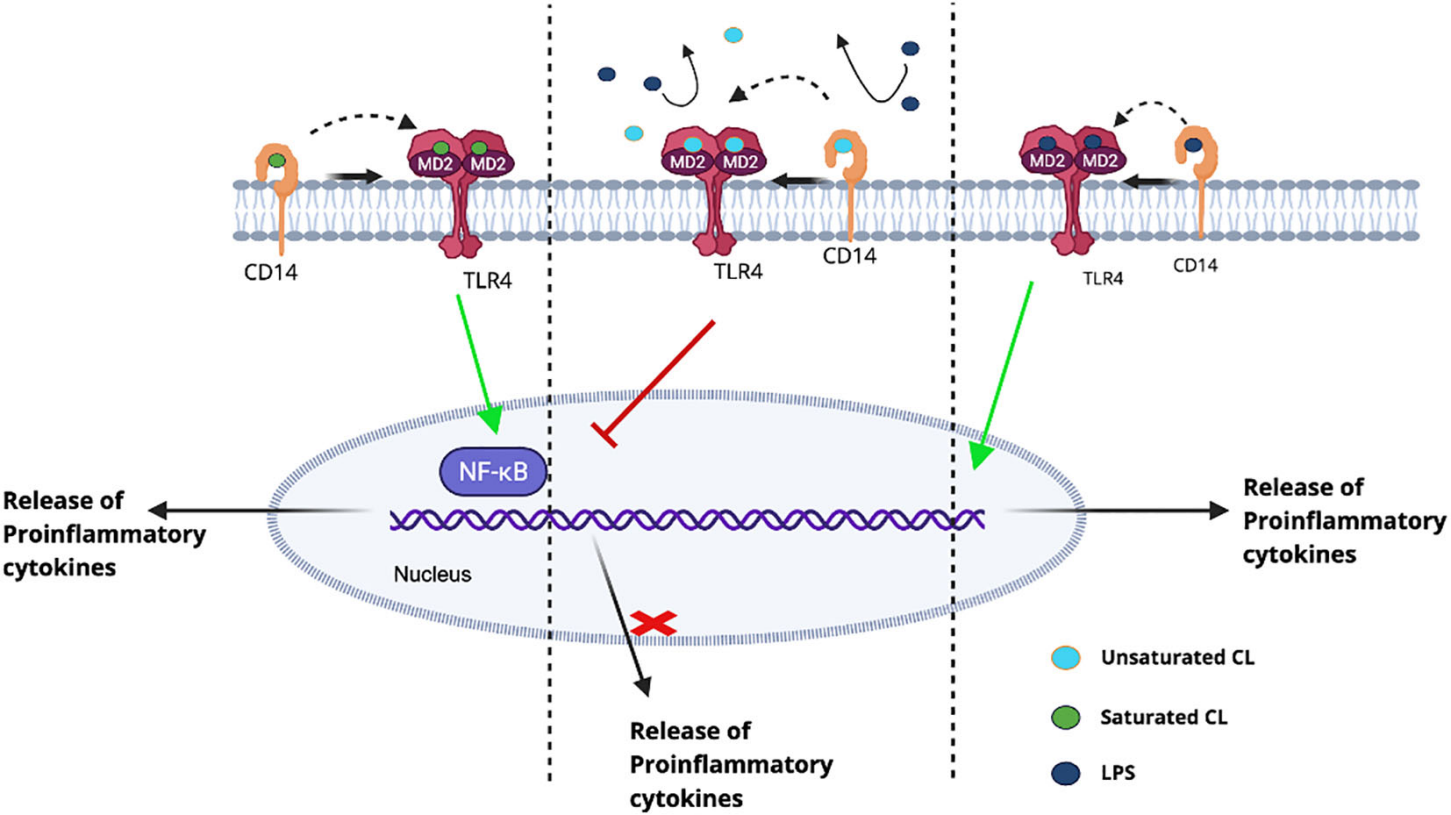
Dual regulatory effects of cardiolipin on TLR4 signaling. LPS binds to the TLR4/MD2 complex, inducing receptor dimerization and triggering the release of proinflammatory cytokines (80). Extracellular unsaturated CL inhibits this pathway by occupying the LPS-binding site on TLR4/MD2, thereby preventing LPS engagement and subsequent signaling. In contrast, saturated cardiolipin directly activates the TLR4/MD2 complex and initiates an inflammatory response (24).

Cardiolipin is also present in the bacterial membrane. In prokaryotes, the acyl chains of cardiolipin are 1–4 carbon atoms shorter than those in eukaryotes and contain mono-unsaturated or saturated acyl chains (75). Cardiolipin and tetra-acylated lipid A share notable similarities in both structure and charge distribution (43). Consequently, extracellular cardiolipin can competitively interfere with cellular responses to LPS (44, 76) (**Figure 2**). This inhibitory effect may resemble that of TLR4 antagonists, unsaturated lipid A derivatives such as Eritoran, which occupy the MD-2 binding pocket without inducing TLR4 dimerization (77). Similar to lipid A, the immunostimulatory activity of cardiolipin is highly structure-dependent. Cardiolipin species containing unsaturated acyl chains exert antagonistic effects on TLR4 signaling. However, as the degree of acyl chain saturation increases, cardiolipin progressively loses its antagonistic properties, and fully saturated cardiolipin functions as a TLR4 agonist (24). This structural dependence may enable immune cells to discriminate between endogenous and exogenous danger signals, as bacterial cardiolipin typically contains highly saturated fatty acids. Activation of the TLR4 pathway by saturated cardiolipin appears to require both MD-2 and CD14, since NF-κB activation is markedly diminished in cells deficient in either molecule (24).

Cardiolipin competitively interrupts the binding of several proteins with LPS, including LPS binding protein (LBP), CD14, and TLR4, thereby reducing the presentation of LPS to MD-2/TLR4 (43, 78). The inhibitory effect of cardiolipin is more pronounced when administered prior to LPS stimulation than during co-stimulation, whereas its addition after LPS exposure fails to elicit inhibition (11, 43). These findings support a mechanism whereby the TLR4/MD-2/LPS complex undergoes internalization following activation, thereby escaping further interaction with extracellular ligands (79).

In conclusion, cardiolipin directly interacts with proteins involved in TLR4 signaling, serving as an agonist or antagonist and regulating inflammatory responses.

#### 2.3.2 TLR2

Cho et al. reported that cardiolipin (purchased from Avanti Polar lipids) activates TLR2 on the surface of antigen-presenting cells (APCs), inducing NF-κB activation, enhancing the expression of co-stimulatory molecules, and promote the activation of naïve T cells (46). This effect appears to be mediated through CD14 rather than MD-2, as the response is abolished in CD14, but not MD-2, deficient cells. *In vivo* experiments using CD14 knockout mice further support the important role of CD14 in cardiolipin-induced immune activation (46). However, the study was limited to APCs and did not examine cytokine transcription or secretion, key downstream events of TLR2 signaling. More recently, two manuscripts from the same research group described the *de novo* synthesis of two types of cardiolipins originally discovered from two different bacteria, *Streptococcus pyogenes* and *Muribaculum intestinale* (81, 82). Using cell-based assay, the studies revealed that these synthetic cardiolipins stimulates the release of TNF-α and IL-23 from macrophages and dendritic cells in a TLR2 dependent manner. Since TLR2 normally functions as heterodimers with TLR1 or TLR6, they further examined the contribution of TLR1 an TLR6, and suggested that TLR1 was involved in cardiolipin included signaling. Notably, these two bacterial cardiolipins possess both unsaturated and saturated acyl chains with defined stereochemistry. This structural specificity is crucial – altering the stereochemistry abolishes their pro-inflammatory activity. These hetero-acylated species complement the findings of Pizzuto et al. (24), which demonstrated that fully saturated or fully unsaturated cardiolipins activate TLR4 rather than TLR2.

### 2.4 NLRP3

**Figure 3:**
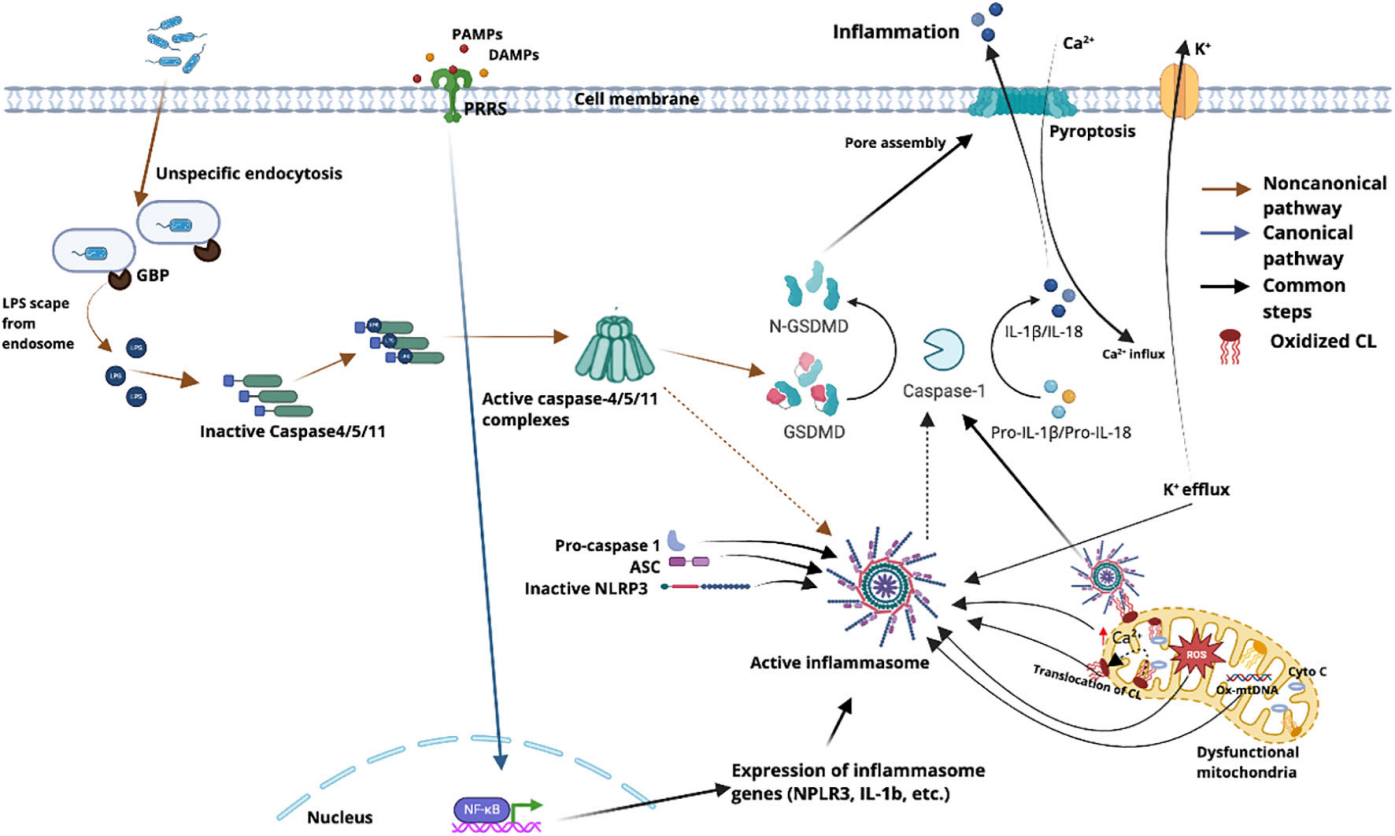
Molecular events linking mitochondrial dysfunction, cardiolipin (CL) dynamics, and inflammasome activation via canonical and non-canonical pathways. In the canonical pathway (blue arrows), pattern recognition receptors (PRRs) detect pathogen-associated molecular patterns (PAMPs) or damage-associated molecular patterns (DAMPs), triggering NF-κB–mediated transcription of inflammasome components, including pro–IL-1β, pro–IL-18, and NLRP3. In parallel, the non-canonical pathway (brown arrows) involves nonspecific endocytosis of lipopolysaccharide (LPS), which escapes the endosome via guanylate-binding proteins (GBPs) and activates cytosolic caspase-4/5/11 complexes. These caspases cleave gasdermin D (GSDMD), generating the pore-forming N-terminal fragment (N-GSDMD) that induces pyroptosis and disrupts ion homeostasis through potassium (K^+^) efflux and calcium (Ca²^+^) influx (96), thereby eliciting cellular stress. This stress contributes to mitochondrial dysfunction, further promoting inflammasome activation. Damaged mitochondria generate reactive oxygen species (ROS), release cytochrome c and oxidized mitochondrial DNA (Ox-mtDNA), and drive the oxidation and translocation of cardiolipin (CL) to the outer mitochondrial membrane. In this context, oxidized CL serves as a platform that recruits and anchors key inflammasome components. By binding to the leucine-rich repeat (LRR) domain of NLRP3, cardiolipin facilitates inflammasome assembly through the recruitment of ASC and pro–caspase-1. Activated caspase-1 subsequently processes pro–IL-1β and pro–IL-18 into their mature inflammatory forms and amplifies pyroptosis through additional GSDMD-mediated pore formation (97, 98). Solid arrows indicate direct activation events, dotted arrows represent indirect or downstream effects (such as NLRP3 activation secondary to caspase-4/5/11), and black arrows denote common downstream steps shared by both pathways.

The NLRP3 inflammasome is a multi-protein complex that plays a central role in innate immunity. Its activation triggers a robust inflammatory response, leading to the processing and secretion of potent pro-inflammatory cytokines, specifically interleukin-1 beta (IL-1β) and interleukin-18 (IL-18) (47, 83). Cardiolipin has been reported to interact with NLRP3, and this interaction has been proposed to be important for the activation of the NLRP3 inflammasome (6, 84) (**Figure 3**).

The assembly of the NLRP3 inflammasome complex typically involves NLRP3, the adaptor protein ASC, and procaspase-1. The direct binding of cardiolipin to NLRP3 has been suggested to promote the assembly of this complex, which in turn leads to the autocatalytic cleavage and activation of caspase-1 (85). Once activated, caspase-1 processes the inactive precursor forms of pro-IL-1β and pro-IL-18 into their mature, biologically active forms, which are then released from the cell, initiating widespread inflammatory responses throughout the organism. NLRP3 can be activated by various stimuli, such as DAMPs, extracellular ATP, pore-forming toxins, viral RNA, particulate matter, and K^+^ transporters (86). Although it is still unclear whether these diverse agonists converge on a single endogenous ligand, reduction of ATP production as a result of mitochondrial damage has been considered a key step (87).

Cardiolipin was first reported in 2013 to bind to the leucine-rich repeat (LRR) domain of NLRP3 (41). Cardiolipin binding has been proposed to promote NLRP3 activation by inducing its conformational changes followed by phase separation (88). Silencing of cardiolipin synthase (CRLS) using siRNA significantly reduces NLRP3-dependent IL-1β release (41, 89). In liver tissues from mice with non-alcoholic steatohepatitis, CRLS knockdown via shRNA also decreased the expression of both CRLS and NLRP3 (90), suggesting that further investigation is needed to determine whether CRLS silencing downregulates NLRP3 expression in general. Additionally, palmitic acid, a known inhibitor of cardiolipin synthesis, has been shown to diminish NLRP3 association with mitochondria, an effect initially attributed to reduced reactive oxygen species (ROS) production (91). Notably, palmitic acid treatment does not alter total NLRP3 levels or TLR/NF-κB pathway activation, supporting an essential role for cardiolipin, rather than ROS, in regulating NLRP3 activation (11).

Subsequent studies revealed that cardiolipin translocates to the OMM upon ROS stimulation, promoting the assembly and activation of the NLRP3 inflammasome on the mitochondrial surface in a Ca²^+^-dependent manner (42). This assembly occurs as early as the TLR-mediated priming stage, during which ROS generated by LPS stimulation appear to promote cardiolipin translocation to the OMM (92). While priming is typically considered to upregulate NLRP3 and pro–IL-1β expression, these findings suggest that NLRP3 activation may begin earlier than previously recognized. Although canonical activators such as nigericin and ATP are generally considered to stimulate NLRP3 via ROS-independent mechanisms, treatment with ROS inhibitors following LPS priming suppresses IL-1β secretion induced by these stimuli (93, 94), indicating that ROS is critical for optimal NLRP3 inflammasome activation. Nonetheless, it remains unclear how ROS-independent NLRP3 agonists, such as linezolid, facilitate cardiolipin binding to NLRP3 in intact cells (41). Direct binding between NLRP3 and cardiolipin has also been demonstrated using isolated mitochondrial fractions (41).

As an inducer of apoptosis, Cyt c has been reported to compete with NLRP3 for cardiolipin binding (95). Conversely, activation of NLRP3 reduces Cyt c release (87), suggesting that cardiolipin may function as a molecular switch between apoptosis and pyroptosis, with the predominance of either pathway ultimately determining the cell’s fate.

Although cardiolipin has been implicated in NLRP3 inflammasome activation, its precise role remains incompletely understood. Owing to its capacity to recruit caspase-1 (see below), cardiolipin has been proposed to act as a scaffold for NLRP3 assembly (84, 99). However, its broad protein-binding capability raises the question of whether cardiolipin functions merely as a structural platform or as an active signaling regulator. Notably, inhibition of cardiolipin synthesis disrupts the mitochondrial electron transport chain, which may secondarily impair NLRP3 activation (100). Aside from NLRP3, cardiolipin has not been reported to interact with other inflammasomes such as AIM2 or NLRC4, and suppression of cardiolipin synthase does not affect their activation. Jonathan et al. proposed an intriguing hypothesis that NLRP3 may act as a sensor of excess acidic lipids, detecting exposed cardiolipin when mitochondrial integrity is compromised and lipid homeostasis is perturbed (101). This model could account for the observed localization of NLRP3 to multiple subcellular compartments and highlights the importance of these associations for inflammasome activation. In summary, the role of cardiolipin in NLRP3 activation remains to be fully defined and warrants further investigation.

### 2.5 Gasdermins

**Figure 4:**
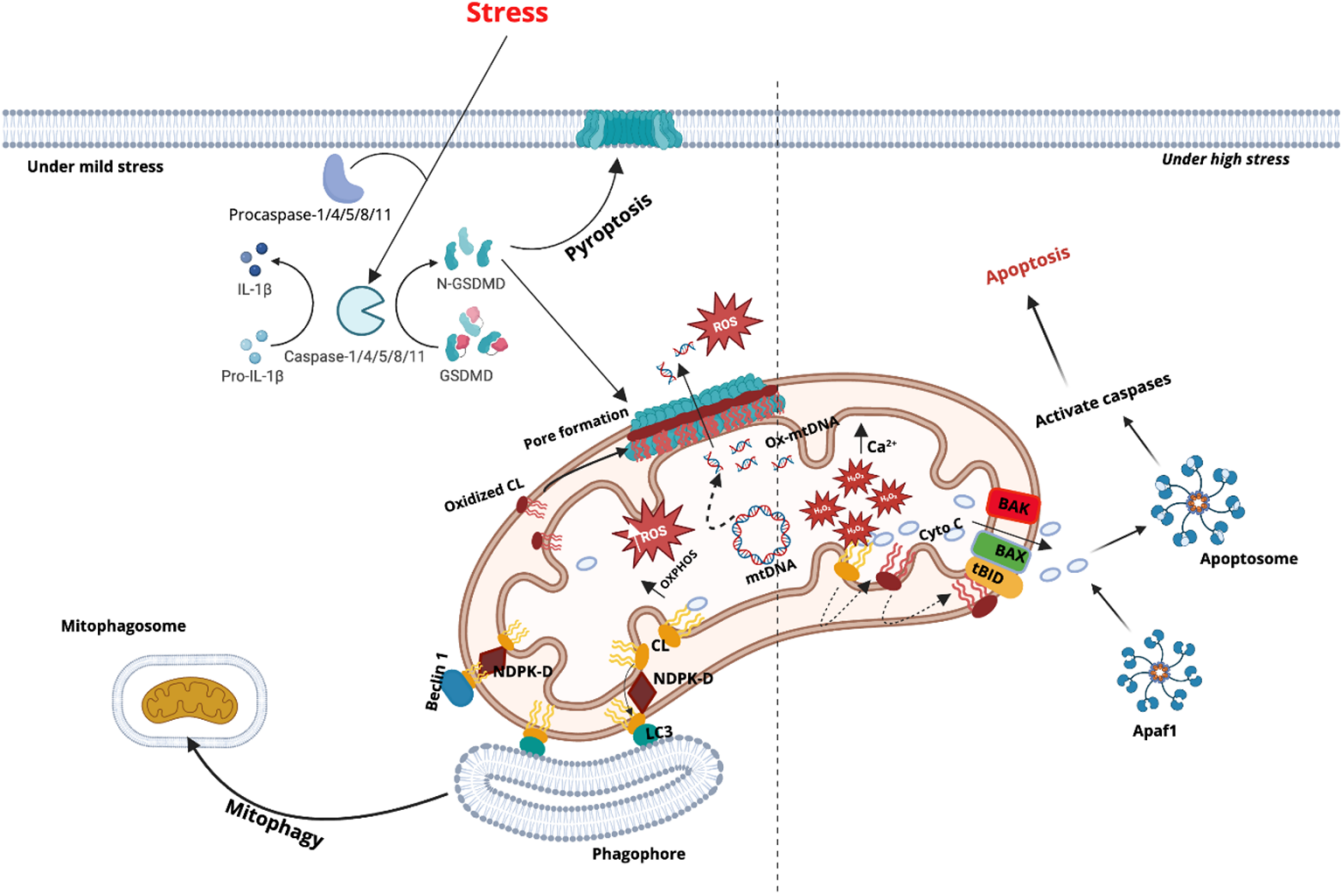
Cardiolipin as a central regulator of mitochondrial fate: pyroptosis, apoptosis, and mitophagy under cellular stress. Under stress conditions, multiple mitochondrial response pathways converge on cardiolipin (CL) signaling. Activation of caspase-1/4/5/8/11 leads to cleavage of gasdermin D (GSDMD), producing the N-terminal fragment (GSDMD-NT), which binds to cardiolipin on the OMM to form transmembrane pores. These pores disrupt membrane integrity, causing release of ROS, oxidized mitochondrial DNA (Ox-mtDNA), and other inflammatory signals. During high stress, excessive ROS—driven by OXPHOS overload and Ca²^+^ accumulation—exacerbate mtDNA damage. Simultaneously, cytochrome c (Cyt c), oxidized by CL in the presence of hydrogen peroxide (H_2_O_2_), translocates to the cytosol. Interaction between translocated CL and tBID induces BAX/BAK oligomerization at the mitochondrial outer membrane, triggering apoptotic membrane permeabilization and cytochrome c release, culminating in apoptosome formation and caspase-dependent apoptosis. In contrast, under mild stress conditions, CL translocates from the inner to the outer mitochondrial membrane with the help of nucleoside diphosphate kinase D (NDPK-D). At the OMM, CL binds LC3A, promoting mitophagy by recruiting phagophores to eliminate damaged mitochondria. Beclin-1 assists in the formation of mitophagosomes that engulf dysfunctional organelles for lysosomal degradation.

Pyroptosis is a lytic form of programmed cell death mediated by the gasdermin family of proteins (66). This family comprises six members—GSDMA, GSDMB, GSDMC, GSDMD, GSDME, and DFNB59—which share high sequence and structural homology, as well as similarities in function and activation mechanism. Most gasdermins play important roles in initiating pyroptosis, with GSDMD and GSDME being the most extensively characterized. Upon inflammasome activation, caspases cleave gasdermins to release their N-terminal fragments, which oligomerize and form pores in the plasma and mitochondrial membranes, thereby compromising membrane integrity and inducing cell lysis (66, 102) (**Figure 4**).

### 2.5.1 GSDMD

Liu et al. first reported that GSDMD-NT was present in cell lysates containing plasma membrane and mitochondrial fractions, and it could bind to cardiolipin-containing membranes and induce leakage of cardiolipin liposomes (47). More recently, Miao et al. discovered that during NLRP3 inflammasome activation, GSDMD-NT bound to cardiolipin present on the OMM and forms pores, leading to mitochondrial damage. This damage led to substantial ROS production, which further promoted cardiolipin exposure in a positive feedback loop, thereby amplifying the inflammatory signal (31, 103). Knockout of cardiolipin synthase or its transporter PLSCR3 inhibited both mitochondrial damage and pyroptosis. Additionally, GSDMD-NT has been found to preferentially target and disrupt mitochondria before affecting the plasma membrane (31). The release of mitochondrial DNA (mtDNA) and mitochondrial ROS (mtROS) further activates related inflammatory pathways (103–105), helping to explain NLRP3 activation observed in the non-canonical inflammasome pathway (106). A similar phenomenon has been observed in apoptotic models. In cardiomyocytes stimulated with poly(I:C) and LPS, caspase-4/11–activated GSDMD-N-terminal fragments preferentially oligomerize on the mitochondrial membrane, as demonstrated by blue native PAGE analysis. At this site, GSDMD interacts with BAX and VDAC1 to form large pores, which may amplify apoptotic signaling and contribute to cardiac dysfunction (107). Inhibition of cardiolipin synthesis or oxidation was found to block pore formation on the mitochondrial membrane, indirectly suggesting cardiolipin as a GSDMD target (107). In the same study, pore formation was shown to be specifically promoted by oxidized cardiolipin (oxCL), which is generated through complex II–mediated oxidation, and inhibition of oxCL production with XJB-5–131 supported its critical involvement in this process (107). Interestingly, NLRP3 was also upregulated in this model, although its role remains unclear.

The binding of GSDMD to cardiolipin was later confirmed using kinetic methods (108). Arumugam et al. used cardiolipin-coated beads to pull down GSDMD-NT and showed that the binding depends on the palmitoylation of GSDMD-NT (109). Furthermore, palmitoylation of GSDMD, induced by increased ROS, has been proposed to facilitate the release of C-terminal inhibition, allowing the full-length GSDMD—normally inactive due to C-terminal autoinhibition—to form pores independently of cleavage (110).

The binding of GSDMD to cardiolipin likely involves electrostatic interactions between acidic phosphate groups of cardiolipin and basic residues at the N-terminus of GSDMD (111). The inner surface of the GSDMD pore formed upon oligomerization is negatively charged, which favors the selective release of mature IL-1β and IL-18—cytokines with relatively low charge density and small molecular size (112).

Given that cardiolipin is present in bacterial membranes, GSDMD-NT may possess direct bactericidal potential. Liu et al. demonstrated that purified GSDMD-NT rapidly killed a range of bacteria, including *Escherichia coli*, *Staphylococcus aureus*, and *Listeria monocytogenes* (113). Notably, both the supernatant from pyroptotic cells and the cells themselves (containing active GSDMD-NT) reduced extracellular and intracellular bacterial loads, respectively, in a GSDMD-NT–dependent manner (47), and similar findings have been observed *in vivo* (111). Cardiolipin is typically localized to the bacterial inner membrane. It remains unresolved how GSDMD-NT gains access to inner membrane cardiolipin or whether bacteria translocate cardiolipin to their outer membrane during infection. Some intracellular bacteria have been reported to externalize cardiolipin during host cell invasion (76, 114), though this is often considered a bacterial survival strategy. Direct visualization of GSDMD-NT binding to bacterial membranes—using electron or fluorescence microscopy—could offer critical mechanistic insights.

#### 2.5.2 Other gasdermins

GSDME can function as a substitute for GSDMD in mediating pyroptosis. In macrophages lacking GSDMD or caspase-1/11, sustained activation of the NLRP3 inflammasome led to the activation of caspase-3 and caspase-8, which subsequently triggered GSDME-dependent pyroptosis (66). Similar to GSDMD-NT, the cleaved N-terminal fragment of GSDME (GSDME-NT) can bind cardiolipin-containing liposomes and induce membrane leakage. Supporting this, GSDME-NT has also been shown to exert toxicity against *E. coli* (115), consistent with earlier observations with GSDMD-NT.

Interestingly, certain chemotherapeutic agents and TNF have been reported to activate caspase-3 to cleave GSDME, leading to a shift from apoptosis to pyroptosis (115). During this process, cardiolipin oxidation by Cyt c, previously associated with apoptosis, still occurred, generating lipid ROS. This finding expands the role of Cyt c–mediated cardiolipin oxidation to include pyroptosis. A recent study further demonstrated that UVC irradiation induced cardiolipin oxidation and translocation to the OMM while simultaneously triggered DNA damage and the release of poly(ADP-ribose) (PAR) polymers. These PAR polymers appeared to induce a conformational change in full-length (FL) GSDME, relieving its autoinhibition and allowing it to execute pyroptosis (116). Although direct binding of FL-GSDME to mitochondrial cardiolipin has not been confirmed, cardiolipin oxidation and exposure appeared to play a critical role in this process.

Additional evidence comes from Shao and co-workers, who found that the N-terminal domains of several gasdermin family members, including GSDME, GSDMA, and the mouse-specific GSDMA3, could bind to cardiolipin-containing liposomes, induce leakage, and lyse *Bacillus megaterium* protoplasts enriched in cardiolipin (102, 117). In contrast, FL gasdermins lacked this activity due to autoinhibition by their C-terminal domains. A notable exception is GSDMB, which does not bind cardiolipin but instead interacts with sulfatide. Moreover, no autoinhibition by the C-terminal domain was reported, likely reflecting unique structural features (118). Interestingly, Justin et al. reported different results: GSDMB-N-terminal fragments were found to bind both cardiolipin and lipid A in liposomes and on the surface of *Shigella flexneri*, mediating bacterial killing without damaging host cells (119). This discrepancy may stem from variations in experimental design, specifically, the use of mixed-lipid liposomes in Justin et al.’s study. These composite membranes, which included multiple phospholipids alongside cardiolipin, may have provided a synergistic binding environment that facilitated GSDMB interaction.

#### 2.5.3 Gasdermin homologs

Gasdermin homologs are conserved across bacteria, fungi, and invertebrates, and all possess the capacity to form membrane pores (120). Among them, only the gasdermin homolog RCD-1 from the filamentous fungus *Neurospora crassa* has been shown to bind cardiolipin. In contrast, bacterial gasdermins (bGSDMs) do not seem to rely on cardiolipin recognition for membrane insertion. Instead, bGSDMs can form pores in membranes with simple lipid compositions, without distinguishing between plasma and organelle membranes—a property likely reflecting the comparatively simple membrane architecture of bacteria (120, 121).

### 2.6 Caspases, orchestrating inflammatory signaling and cell death pathways

#### 2.6.1 Caspase-1

*Eric* et al. reported that caspase-1 bound to cardiolipin and underwent ROS-dependent oligomerization independently during the priming stage of inflammasome activation (42). Caspase-1 oligomerized when incubated with cardiolipin-containing liposomes, as proved by the formation of high-molecular-weight protein complexes from the bissulfosuccinimidyl suberate (BS3) crosslinking assay. Thus, ROS likely induced cardiolipin externalization and the subsequent caspase-1 oligomerization. Cardiolipin appeared to recruit both NLRP3 and caspase-1, facilitating the spatial assembly of ASC and other inflammasome components, thereby accelerating activation of the NLRP3 inflammasome (122).

Caspase-1 has not been shown to bind other phospholipids. Moreover, although caspase-1 functions downstream of multiple inflammasomes, there is currently insufficient evidence to suggest that cardiolipin participates in the activation of inflammasomes other than NLRP3. These differences may reflect distinct subcellular localizations of the complexes. Notably, caspase-1 binding to cardiolipin appears to occur independently of NLRP3. Caspase-1 has been shown to interact directly with isolated cardiolipin in a dose-dependent manner (42), although its precise binding site remains unidentified. The interaction is likely mediated through a combination of electrostatic and hydrophobic interactions.

#### 2.6.2 Caspase-4/11

The non-canonical inflammasome pathway is mediated by caspase-4/11 sensing of cytosolic LPS. Studies using cell lysates have shown that cardiolipin interacted with caspase-11 and promoted its oligomerization (as suggested by the molecular shift from the BS3 crosslinking assay) (42). Given the structural similarity between cardiolipin and lipid A, this interaction is biologically plausible. However, whether caspase-11 binds to mitochondrial cardiolipin under physiological conditions, and the functional relevance of such binding, remains to be fully elucidated.

**Figure 5:**
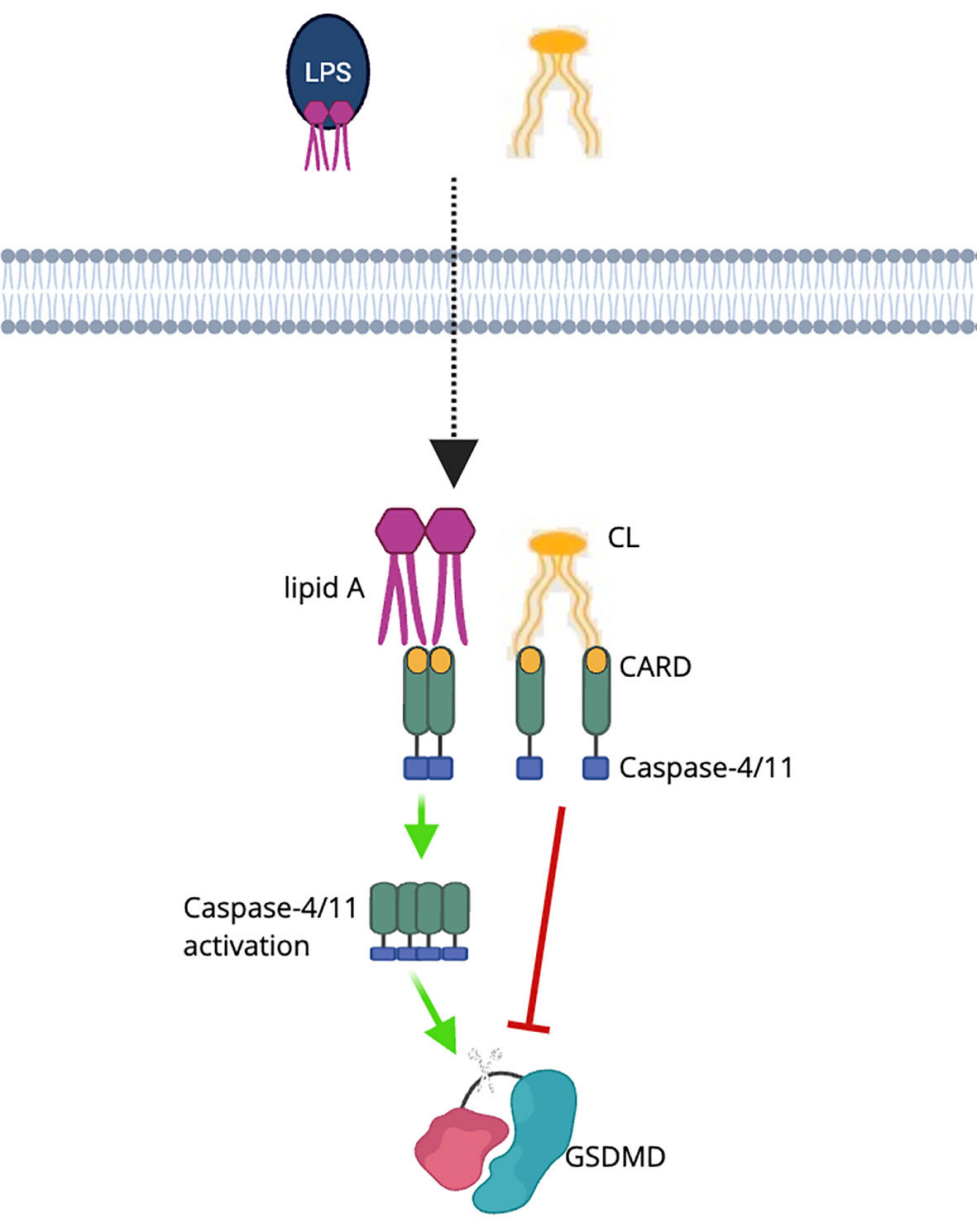
Cardiolipin inhibits caspase-4/11 activation in the non-canonical inflammasome pathway. In the cytosol, LPS, through its lipid A component, directly binds to the CARD domain of caspase-4/11, promoting oligomerization and activation. This activation leads to GSDMD cleavage and pyroptotic cell death. Cardiolipin (CL) competes with lipid A for binding to the CARD domain, thereby blocking LPS-induced caspase-4/11 oligomerization and inhibiting downstream GSDMD activation. This mechanism highlights the anti-inflammatory role of cardiolipin in modulating the non-canonical inflammasome pathway (25).

Recently Pizzuto et al. reported that exogenous cardiolipin containing 18:2 acyl chains could bind to the CARD domain of caspase-4/11, thereby inhibiting LPS-induced activation of the non-canonical inflammasome. This binding suppressed GSDMD cleavage and IL-1β release, ultimately alleviated endotoxemia in mice (25). Interestingly, once internalized by cells, this exogenous cardiolipin localizes to the OMM (25). Despite this localization, the additional cardiolipin did not seem to promote binding of NLRP3, GSDMD, or caspase-1, suggesting certain levels of specificity in cardiolipin–caspase-4/11 interactions (**Figure 5**).

#### 2.6.3 Caspase-8

Caspase-8—primarily known for its role in apoptosis—has also been reported to bind cardiolipin, but the structural basis for this interaction has yet to be elucidated (48). Gonzalvez et al. showed that caspase-8 interacted with mitochondria via cardiolipin. During apoptosis, cardiolipin translocates from the IMM to OMM, potentially serving as an anchoring site for caspase-8, enabling its direct localization to mitochondria (48, 50). At the mitochondrial surface, caspase-8 effectively cleaves Bid, a member of the Bcl-2 protein family, into its truncated active form (tBid) (48, 49). The formation of the caspase-8, cardiolipin, and Bid complex has been suggested to promote OMM permeabilization, leading to Cyt c release and initiation of the intrinsic apoptosis cascade (48, 50). These studies suggest a direct link between the extrinsic and intrinsic apoptotic pathways, as well as a role for cardiolipin as a functional platform for apoptosis signaling (48, 123).

### 2.7 pro-IL-1α

Dagvodprj et al. reported that pro-IL-1α, the inactive form of the inflammatory cytokine IL-1α, binds directly to cardiolipin in a Ca^2+^ dependent manner (124). Pro-IL-1α features an LC3-interacting region (LIR) motif and is proposed to interact with cardiolipin similarly as those autophagy-related proteins. Thus, pro-IL-1α is found to compete with LC3 for cardiolipin binding, prevent the clearance of damaged mitochondria and subsequently promote the activation of the NLRP3 inflammasome (124).

## 3 Summary and future perspectives

Cardiolipin, a distinctive phospholipid of the mitochondrial membrane, is increasingly recognized as a central and versatile signaling molecule that profoundly influences immune responses. As illustrated by the examples discussed above, its interactions with a wide range of proteins appears to be essential for both initiating and fine-tuning immune processes. For example, cardiolipin has been reported to directly interact with innate immune sensors such as the NLRP3 inflammasome (41), triggering robust inflammatory cascades. Concurrently, its interactions with LC3 and Beclin 1 have been implicated as key signals for mitophagy (39, 40), facilitating the removal of damaged organelles and preventing chronic inflammation.

### 3.1 Controversies and knowledge gaps

Despite substantial progress in elucidating cardiolipin’s immunological roles, many fundamental questions remain unanswered. The precise molecular mechanisms underlying cardiolipin–protein interactions in immune cells, including the specific binding sites, conformational rearrangements upon binding, and downstream signaling events, require further clarification. A key direction for future research is to determine how different cardiolipin species, particularly those with distinct fatty acyl chain compositions, modulate specific immune signaling pathways.

Cardiolipin oxidation and externalization are generally regarded as coupled for its function in danger signaling (1). However, this paradigm is being challenged. For example, stimulation with actinonin, rotenone, or 6-hydroxydopamine has been shown to induce cardiolipin translocation to the OMM without oxidation (125). During apoptosis, cardiolipin in the intermembrane space is oxidized by Cyt c, yet its binding to caspase-8 appears to occur independent from oxidation (75). Moreover, most studies employing cardiolipin-containing liposomes utilize non-oxidized species. Whether cardiolipin undergoes oxidation during inflammasome activation—and the functional implications of such modification—remains to be determined. Technical challenges in analyzing cardiolipin’s acyl chain composition and oxidative status (126), as well as limited data on the kinetics of its protein interactions, continue to hinder a deeper understanding of its role in membrane-mediated signaling.

Recent studies have highlighted the importance of palmitoylation in NLRP3 inflammasome activation (88, 109, 110). Interestingly, palmitoylation exerts distinct effects on NLRP3 and GSDMD: it enhances the affinity of GSDMD-NT for cardiolipin, promoting mitochondrial recruitment and pore formation (109), while decreases NLRP3 solubility, facilitating phase separation and inflammasome assembly (88). This post-translational modification may modulate protein conformation in a manner analogous to binding with a lipid. Exploring the interplay between cardiolipin binding and palmitoylation—and their potential synergistic contributions to inflammasome activation—may uncover new mechanistic insights and help explain cell-type-specific variability in inflammasome function.

Most current evidence for cardiolipin–protein interactions comes from studies using purified proteins, liposomes, or isolated mitochondria. However, direct evidence of these interactions in live cells under physiological conditions remains lacking. In addition, cardiolipin-containing liposomes are highly prone to oxidation, making it difficult to control their actual lipid composition (127). Therefore, it cannot be ruled out that some observed proteins binding occurs with oxCL generated during the liposome preparation.

### 3.2 Therapeutic potentials

Cardiolipin plays a fundamental role in maintaining mitochondrial structure and function by preserving cristae integrity and supporting the activity of electron transport chain complexes (1, 128). Consequently, cardiolipin abnormalities—such as peroxidation—are closely associated with various mitochondrial diseases (129–131). Several therapeutic strategies have been developed that directly or indirectly target cardiolipin to treat mitochondria-related disorders.

Elamipretide (SS-31) is a synthetic tetrapeptide capable of penetrating cell membranes and selectively accumulating in the IMM, where it appears to bind cardiolipin via electrostatic interactions (132). This interaction is believed to prevent cardiolipin peroxidation, preserve mitochondrial cristae structure, enhance electron transport chain activity, and inhibit apoptosis. In clinical studies, Elamipretide has been shown to improve symptoms of Barth syndrome, a rare X-linked disorder caused by mutations in *Tafazzin* (TAZ), by significantly increasing skeletal muscle strength and cardiac stroke volume (133) (21, 134). In 2025, Elamipretide (Forzinity^®^) received FDA accelerated approval as the first treatment for Barth syndrome in patients weighing at least 30 kg (135). Beyond Barth syndrome, Elamipretide has demonstrated potential in preserving photoreceptor function and slowing vision loss in age-related macular degeneration (AMD), likely through reducing oxidative stress and apoptosis, thereby improving the survival of retinal endothelial, trabecular meshwork, and retinal pigment epithelial cells (136). Although Elamipretide improves mitochondrial function and mitigates organ injury in animal models of heart failure, diabetic kidney disease, and neurodegeneration, several clinical trials have failed to meet their primary efficacy endpoints (132, 137–139). These discrepancies underscore the need for more precise patient selection and disease stratification in future studies. Despite these challenges, Elamipretide remains a promising therapeutic candidate for disorders associated with mitochondrial dysfunction.

Bevemipretide (SBT-272) is a blood–brain barrier–penetrant compound developed for neurodegenerative diseases characterized by mitochondrial dysfunction and TDP-43 pathology (140). Bevemipretide is proposed to stabilize cardiolipin to preserve mitochondrial structure and function, enhance mitochondrial transport along axons, reduce glial activation, and reduce TDP-43 aggregation (140). In preclinical amyotrophic lateral sclerosis (ALS) models, Bevemipretide improved motor performance and mitochondrial integrity (140, 141). Phase 1 clinical trials have demonstrated its safety, and the compound has received FDA Orphan Drug designation for ALS, highlighting its translational potential (141). In addition, topical ocular administration has shown retinal protective effects in age-related macular degeneration (AMD) models (142). Bevemipretide remains in early clinical development, and further studies are needed to evaluate its efficacy across a broader range of mitochondrial disorders.

XJB-5-131, a mitochondria-targeted electron scavenger, has been shown to improve behavioral outcomes in mouse models of traumatic brain injury, presumably by inhibiting cardiolipin peroxidation (143). However, prolonged or high-dose administration may disrupt physiological ROS-dependent mitochondrial functions. Moreover, its blood–brain barrier permeability and clinical efficacy in humans have yet to be established.

CMP3013, a cyclohexylamine-containing, cell-penetrating α-helical amphipathic peptide, exhibits high affinity for cardiolipin (144). In a mouse model of acute kidney injury, CMP3013 preserved mitochondrial cristae structure, reduced ROS generation, enhanced ATP production, and restored renal function. Nevertheless, as a peptide-based therapeutic, its stability and sustained efficacy under chronic or long-term treatment conditions warrant further investigation (144).

More recently, ABHD18 has been identified as a novel therapeutic target for Barth syndrome caused by TAZ loss-of-function mutations (145). TAZ deficiency impairs cardiolipin remodeling, resulting in reduced levels of mature cardiolipin and accumulation of immature intermediates. ABHD18 catalyzes the conversion of mature cardiolipin into monolysocardiolipin. Inhibition or genetic knockout of ABHD18 in cellular and animal models increased mature cardiolipin levels and improved mitochondrial structure and function. However, as a member of the lipase family, potential off-target effects on global lipid metabolism remain a concern (145, 146).

Cardiolipin and its analogs also hold promise in drug delivery applications. Owing to its amphipathic structure, high negative charge, and intrinsic mitochondrial affinity, cardiolipin has been increasingly utilized in nanocarrier systems—including liposomes and polymeric micelles (147–150). These nanoscale formulations can enhance drug solubility, bioavailability, and tumor targeting (151–153). In addition, cardiolipin is thought to promote membrane curvature and the formation of high-surface-area structures, thereby increasing drug encapsulation capacity.

Expanding insights into cardiolipin–protein interactions in immune regulation are opening new avenues for therapeutic intervention. The development of pharmacological agents that selectively modulate specific cardiolipin–protein interactions, or cardiolipin-mimetic compounds, holds promise for mitigating pathological inflammation, modulating autoimmune responses, and enhancing immune cell function. Collectively, these emerging strategies may represent a paradigm shift in the treatment of diseases at the interface of mitochondrial dysfunction and immune dysregulation.
